# On the Mode of Manufacturing Sugar-Coated Pills and Granules

**Published:** 1867-06

**Authors:** Henry C. Archibald


*On the Mode of Manufacturing Sugar-Coated Pills and Granules.* By Henry C. Archibald. (An Inaugural Essay, presented to the Philadelphia College of Pharmacy.)

The manufacture of sugar-coated pills and granules having of late become a source of great profit and trade to the apothecary, the mode of manufacturing them being kept secret, and the views advanced by some of our leading pharmacuetists being wholly inadmissible in preparing them, I have, from long practical experience in their manufacture, determined to make it a subject for an essay. In order to make a pill that shall medicinally come up to the standard of the U. S. Pharmacopœia in therapeutic effects, the greatest care requisite in their manufacture is in the selection of the drugs that enter into their composition; for that purpose it is advisable, when you manufacture them largely, to buy the crude drugs, and from them prepare extracts, powders, &c., so as to insure the reliability of the pills, and to keep up for them the reputation they so richly deserve if properly prepared.

The first step in the process of manufacturing pills is essentially as follows: Sufficient mass is made up at one time to be capable of being divided into 2000 pills, great care being observed to have it of sufficient hardness and tenacity to insure the pills after formation against indentation by pressure and crumbling into irregular pieces; after which the mass is rolled between two boards, the upper with teeth inserted for cutting the mass, the bottom one having a guage attached to the sides so as to regulate the sides to suit the mass to be sub-divided previous to rolling them out into pills, and, further, to insure accuracy, each sub-divided piece of mass is carefully weighed on well balanced scales, thereby preventing the possibility of any pill being larger than another. The pills are then cut by machinery suited to the size of the pill, and as they are formed roll into large shallow trays filled with some inert powder, which acts not only as an absorbent of the moisture in the pill, but prevents them, while drying, from becoming irregular and losing their shape. I would state that the trays vary in size and are capable of holding from 7 to 20,000 pills when spread evenly over the surface. When filled, the trays are removed and kept in a heated room, the temperature of which is regulated as nearly as possible to from 80 to 90° F.; when of sufficient hardness they are separated from the powder by sifting, and a coating of a solution of warm gelatine is placed over them, to prevent their adhering together. After the gelatine has thoroughly fixed itself upon the pills, they are thrown into a large circular copper pan, suspended over a fire by means of chains attached to the ceiling, and a thick syrup, made in the proportion of 2 lb av. of sugar to ℥xii. of water, is added successfully with constant attention until dry, and so on until the pills assume a neat and regular appearance. The time it takes to coat pills properly varies much according to their nature; those composed of resins which become soft by heat it takes a longer time, from the fact that you have to lower the temperature of the fire, and consequently a longer time is required to drive off the water in the syrup; but, from experience, I can safely say that the average time consumed to coat properly a batch of 7,000 pills is from 9 to 10 hours. As thus prepared the sugar crystallizes regularly upon the pill and presents to the eye not only a uniform but a smooth appearance; they are entirely soluble and will keep for an indefinite period without becoming hard, and consequently more or less insoluble in the gastric juices of the stomach. I present herewith some compound cathartic pills, together with granules of morphia made and coated by the above process, and have been on hand about four months.

Granules are made upon the same principle, by incorporating the alkaloids or salts with some inert powder and gum arabic for its adhesiveness, and are dried and coated in the same way. I could still further enlarge upon the above process, but my sole aim is to present in as brief a manner as possible only the chief points in their mode of manufacture. —*American Journal of Pharmacy.*

